# Hyaluronic Acid Derivative Effect on Niosomal Coating and Interaction with Cellular Mimetic Membranes

**DOI:** 10.3390/molecules26113434

**Published:** 2021-06-05

**Authors:** Patrizia N. Hanieh, Jacopo Forte, Chiara Di Meo, Maria Grazia Ammendolia, Elena Del Favero, Laura Cantù, Federica Rinaldi, Carlotta Marianecci, Maria Carafa

**Affiliations:** 1 Dipartimento di Chimica e Tecnologie del Farmaco, Sapienza Università di Roma, Piazzle A. Moro 5, 00185 Roma, Italy; patrizianadia.hanieh@uniroma1.it (P.N.H.); jacopo.forte@uniroma1.it (J.F.); chiara.dimeo@uniroma1.it (C.D.M.); maria.carafa@uniroma1.it (M.C.); 2 Centro Nazionale Tecnologie Innovative in Sanità Pubblica, Istituto Superiore di Sanità, Viale Regina Elena 299, 00162 Rome, Italy; maria.ammendolia@iss.it; 3 Laboratorio Interdisciplinare Tecnologie Avanzate (L.I.T.A.), Department of Medical Biotechnologies and Translational Medicine, University of Milan, Via Fratelli Cervi 93, 20090 Segrate, Italy; elena.delfavero@unimi.it (E.D.F.); laura.cantu@unimi.it (L.C.)

**Keywords:** niosomes, HA-Chol derivative, tumor targeting, mimetic membranes, fluorescence, TEM, SAXS

## Abstract

Hyaluronic acid (HA) is one of the most used biopolymers in the development of drug delivery systems, due to its biocompatibility, biodegradability, non-immunogenicity and intrinsic-targeting properties. HA specifically binds to CD44; this property combined to the EPR effect could provide an option for reinforced active tumor targeting by nanocarriers, improving drug uptake by the cancer cells via the HA-CD44 receptor-mediated endocytosis pathway. Moreover, HA can be easily chemically modified to tailor its physico-chemical properties in view of specific applications. The derivatization with cholesterol confers to HA an amphiphilic character, and then the ability of anchoring to niosomes. HA-Chol was then used to coat Span^®^ or Tween^®^ niosomes providing them with an intrinsic targeting shell. The nanocarrier physico-chemical properties were analyzed in terms of hydrodynamic diameter, ζ-potential, and bilayer structural features to evaluate the difference between naked and HA-coated niosomes. Niosomes stability was evaluated over time and in bovine serum. Moreover, interaction properties of HA-coated nanovesicles with model membranes, namely liposomes, were studied, to obtain insights on their interaction behavior with biological membranes in future experiments. The obtained coated systems showed good chemical physical features and represent a good opportunity to carry out active targeting strategies.

## 1. Introduction

Cancer remains a main threat for health and one of the leading causes of death in the world. Standard treatments often do not effectively control cancer development and progression. The main issues about cancer therapy are the side effects induced by drugs, their low biodistribution, and their physical-chemical instability. Drug delivery systems (DDS) represent a possible strategy to overcome these limitations in order to obtain a specific drug localization. Nanotechnology-based approach is one of the promising strategies in cancer fighting due to its ability to increase drug delivery localization inside the tumor cells thus reducing the side effects in normal cells [1]. In fact, an appropriate design of the DDS is useful to obtain a targeted therapy and substantially improved outcomes. In this regard, nanocarriers have been intensively studied as DDS for targeting tumors. Hard and soft nanocarriers have been both prepared and characterized and their surface modification or addition of stimuli responsive moieties have emerged as alternatives for more effective cancer treatment [2,3,4].

The first widely used nanocarrier in the market is Doxil (doxorubicin-loaded liposomes) useful for cancer treatment because it is able to decrease doxorubicin cardiotoxicity.

Among the several available nanocarriers, niosomes (Nio), bilayer-structured vesicles obtained by surfactants and cholesterol in an aqueous phase [5], can provide several advantages compared to other DDS in terms of stability, capability to be functionalized, biocompatibility, and lower costs. In order to obtain drug release inside tumor tissue an ideal nanocarrier should simultaneously show high tumor accumulation and cellular internalization.

The surface modification of niosomes can improve their target specificity as cancer drug delivery systems [6,7].

Hyaluronic acid (HA) is one of the most used biopolymers in the development of drug delivery systems, due to its biocompatibility, biodegradability, non-immunogenicity, and its intrinsic targeting properties. 

HA is the main component of extracellular matrix that contributes to several cellular responses such as angiogenesis, cell signaling, tissue structure, wound healing, and tissue hydration [8,9]. Moreover, it is found in the eye vitreous humor, synovial fluid, and in connective tissue [10]. HA has been increasingly applied in medicine as advanced biomaterial in the development of medical devices and drug delivery systems since its discovery. The specific binding of HA to CD44 and the possibility of exploiting the EPR effect could provide an option for nanocarrier active tumor targeting, allowing enhanced cancer cell uptake via the HA-CD44 receptor-mediated endocytosis pathway [11].

After in vivo administration nanocarriers are coated by different biological molecules, such as nucleic acids, cytokines, amino acids, or proteins, which coat the carrier surface. This surface shell is mainly composed of proteins, called “protein corona” (PC). The PC adsorption on nanocarrier surface can affect its physical-chemical properties such as dimensions and ζ-potential that could influence its in vivo behavior and hence its toxicity, in vivo clearance, cellular uptake, cells interaction, and immune response [12]. Understanding the factors related to the protein corona interaction, it is possible to take advantage of this phenomenon to optimize carrier design, ameliorate its efficiency, targeting and blood residence time. The composition of protein corona shell depends on the chemical nature, size, shape, and surface charge of the nanocarrier [13].

However, when nanocarriers are coated with anionic polysaccharides, such as HA or alginate, both protein adsorption and the rate of macrophage uptake decreased. These effects have been connected to the presence of HA coating on nanocarrier surface and hence to the presence of a negative charge that reduces protein adsorption and allows for targeted delivery to cells bearing CD44 receptors as opposed to non-specific cell-uptake mechanisms of the plain nanoparticles [14].

Moreover, HA can be easily chemically modified in order to tailor its physical-chemical properties to make it suitable for the required applications. The derivatization of HA chains with a proper amount of hydrophobic moieties, as cholesterol [15,16] or riboflavin [17], affects solubility of the polymer, which acquires amphiphilic properties. The degree of derivatization and the choice of the hydrophobic moiety can modulate the hydrophobic/hydrophilic balance of the product: with a high derivatization degree of the chains, insoluble structures, i.e., nanohydrogels, can be obtained and used as nanocarriers in drug delivery applications [18,19].

In this work, we prepared a derivative of HA with cholesterol (HA-Chol) with a low degree of derivatization (10% mol_Chol_/mol_HA_), in order to maintain the water solubility of the product and to give it the ability to modify niosomal surface. 

**Figure 1:**
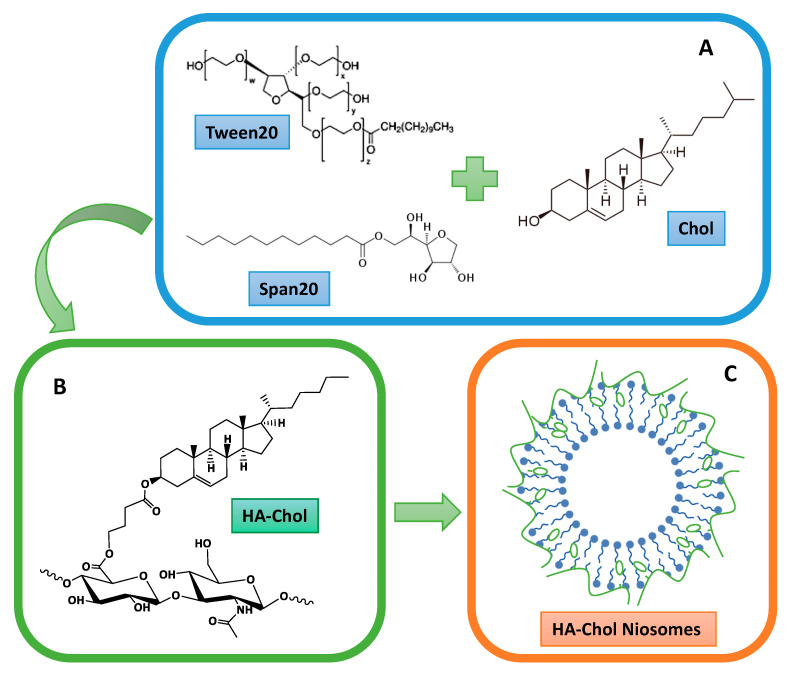
Scheme of HA-Chol-coated niosomal structure, where (**A**) shows the molecular structure of each niosomal compound; (**B**) shows the molecular structure of HA-Chol; (**C**) shows the hypothetical niosomal organization after the preparation.

Span^®^ or Tween^®^ niosomes were prepared and different niosomal formulations were obtained by functionalizing their surfaces by adding the HA-Chol derivative (Figure 1).

The nanocarrier physical-chemical characteristics were analyzed in terms of hydrodynamic diameter, ζ-potential, SAXS, and bilayer features to evaluate the difference between the HA-niosomes and the non-HA ones. Stability studies have been performed over time and in bovine serum. 

Moreover, interaction properties of coated nanovesicles with model membranes, liposomes, were evaluated, to obtain insights on their interaction behavior with biological membranes.

## 2. Results and Discussion

The derivatization of HA with cholesterol moieties (at 15–20% mol_Chol_/mol_HA_) was previously used to obtain insoluble derivatives, able to form nanosized structures when treated with appropriate physical-chemical processes, as nanoprecipitation or autoclave treatments [20]. In that case, however, the proper interaction of the polymer with the niosomes would be difficult and not effective. For this reason, a derivatization degree of 10% mol_Chol_/mol_HA_ was chosen, in order to obtain HA water-soluble product bearing cholesterol moieties able to insert in the external layer of niosomes, anchoring hyaluronan chains on the surface and thus providing a hyaluronan coverage shell.

Mean diameter, polydispersity index (PDI), and ζ-potential measurements were investigated to characterize niosomal structures and to evaluate the presence of HA-Chol on niosomal surface. Uncoated span-based niosomes show a hydrodynamic diameter smaller than 200 nm, even smaller than 100 nm in presence of equal mole fraction of cholesterol. ζ-potential values are all sufficiently negative to assure a good stability of the samples. PDI values confirm that the samples are monodisperse. Uncoated Tween-based niosomes show a diameter under 200 nm and a ζ-potential value negative enough to assure a good stability of the sample. PDI values confirm that the sample is monodispersed (Table 1).

The addition of HA-Chol to the solutions gives rise to an increase in the size of all niosomal preparations. The measured mean hydrodynamic diameter enlarges as a function of HA-Chol fraction. The polydispersity of the systems increases, as expected, while ζ-potential reaches the same negative value in all samples, about −37 mV [21]. Results demonstrate that HA-Chol interacted with niosomes, anchoring to their surface (Table 2).

The Span-based system with the higher content of cholesterol was discarded because it is not possible to obtain upon HA-Chol addition a stable and monomodal population of niosomes [22].

**Figure 2:**
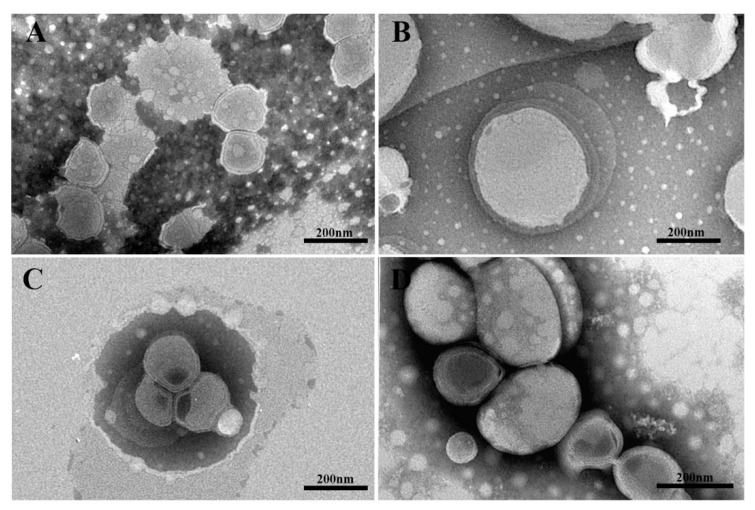
TEM micrographs of representative niosomes negatively stained with sodium phosphotungstate. Uncoated T niosomes (panel (**A**)) and HA-coated Tween 20 niosomes (Panel (**B)**, sample T 1:1). Uncoated S3 (Panel (**C**)) and HA-coated Span-based niosomes (Panel (**D)**, sample S3 1:0.5).

TEM images in Figure 2 showed representative samples of Tween 20- (panels A and B) and Span 20-based (panels C and D) niosomes. The average diameter of the niosomes was found to be well correlated with data obtained from DLS measurements, although the size measurements determined by TEM are smaller than in DLS due to the lack of hydration. Tween 20 niosomes showed an almost spherical shape with a monolamellar shell, whereas those achieved with Span 20 appeared as spherical vesicles with a multilamellar shell. The HA coating does not modify niosome morphology but only their sizes. Partially collapsed structure of some Span 20 niosomes after drying clearly highlighted the presence of a multilayered shell and a hollow structure.

The effective niosome coating with hyaluronan shell, provided by the HA-Chol derivative, was confirmed by NMR analysis in solid state, in particular using the ^13^C CP-MAS (cross-polarization magic angle spinning) NMR technique, which permits to enhance the signals of dilute nuclei (with a low natural abundance and a low gyromagnetic ratio γ), such as ^13^ C, by transferring the magnetization from abundant nuclei, such as ^1^H. For this reason, however, the ^13^C CP-MAS NMR is not a quantitative analysis, because the signal intensities are strictly dependent on the chemical environment of the ^13^C atoms [23]. 

**Figure 3:**
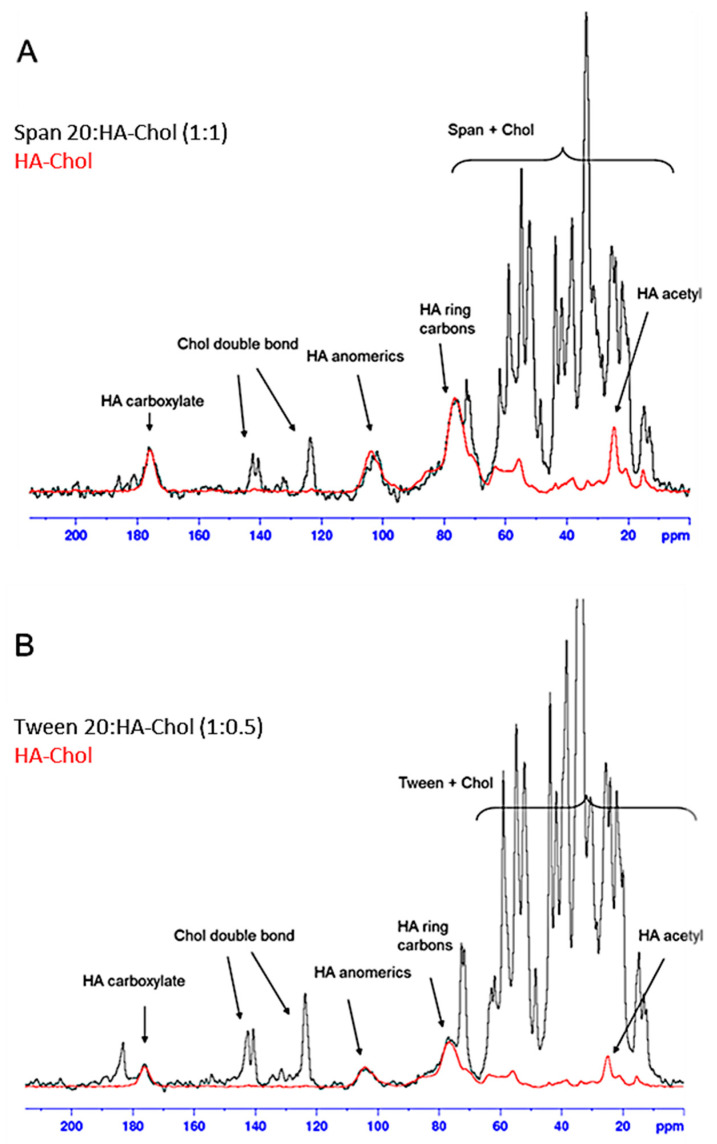
^13^C CP-MAS NMR spectra of the samples S2 1:1 (**A**) and T 1:0.5 (**B**) superimposed with the spectrum of HA-Chol polymer carried out at 200.63 MHz at 27 °C.

The ^13^C CP-MAS NMR spectra of two of the coated niosomes, S2 (with Span 20:HA-Chol in the ratio 1:1) and T (with Tween 20:HA-Chol 1:0.5), were recorded and superimposed with the spectrum of the HA-Chol polymer (Figure 3A and Figure 3B, respectively).

It is possible to identify the characteristic signals of HA (e.g., anomeric carbons, carboxyl group) [24,25], together with the double bond of cholesterol and the overlapping signals of the surfactant and cholesterol aliphatics.

The presence of the coating on niosomes is thus confirmed, and from a qualitative point of view it is possible to highlight how, compared to the surfactant signals, in the sample S2 (1:1) the signals of the polymer are more intense than those found in the sample T (1:0.5).

In order to better understand the interaction of HA-Chol with niosomes and in particular with vesicle bilayer, fluorescence studies were carried out. The effects of HA-Chol on niosome bilayer can be better characterized by using fluorescent probes sensitive to fluidity, microviscosity, and polarity variations inside the bilayer. Obtained results are shown in Table 3. The addition of HA-Chol to niosomal bilayer causes an increase in anisotropy values demonstrating an increase in bilayer fluidity, more evident at the molar ratio 1:1. At the same time, as evidenced by Pyrene experiments, the addition causes also a decrease in polarity, probably due to the increase of cholesterol content in the bilayer and due to a slight decrease in microviscosity; result that confirms the increase of fluidity evidenced by DPH experiments [26].

**Figure 4:**
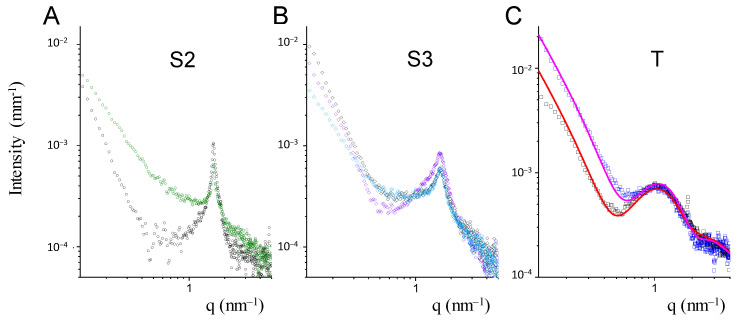
SAXS intensity spectra of Span-based and Tween-based niosomes. Panel (**A**) S2 sample without (black dots) and in presence of HA-Chol (1:1) molar ratio (green dots). Panel (**B**) S3 sample without (black diamonds) and in presence of HA-Chol (1:0.5) molar ratio (light blue diamonds) and (1:1) molar ratio (violet diamonds). Panel (**C**) T sample without (black squares) and in presence of HA-Chol (1:1) molar ratio (blue squares). Lines are the fits with a bilayer form factor.

The internal structure of coated and uncoated niosomes was investigated by small angle X-ray scattering (SAXS). The intensity profiles are reported in Figure 4. The form factors of niosomes have been reconstructed as closed bilayers, multi-lamellar when Span-based, while unilamellar when Tween-based, which are in agreement with the TEM results (Figure 2). 

For both S2 and S3 Span-based niosomes, an intensity peak at q = 1.6 nm^−1^ was revealed, corresponding to the same characteristic internal distance d = 3.9 nm. The layered structure on the local scale is made up of several concentric bilayers in close contact [27]. The amount of cholesterol influences the compactness of the Span bilayers, favoring a more ordered multilayered structure in S2 with respect to S3, with a sharper characteristic peak at q = 1.6 nm^−1^. The insertion of HA-Chol does not seem to affect the peculiar internal arrangement of the Span niosomes. Rather a change in the intensity spectra can be seen in the q < 1 nm^−1^ region, compatible with a change in the electron density of the surface layer, due to HA-Chol insertion in the external layer of the vesicle “membrane.” 

The percentage of surface available for the insertion of HA-Chol molecules in the multilayered structure is less than in unilamellar niosomes. A certain amount of HA-Chol might have not interacted with the surface.

Tween20-based niosomes present a unilamellar structure. Figure 4 reports the intensity profiles of naked and coated niosomes, in a q range (0.1 nm^−1^ ≤ q ≤ 5 nm^−1^) corresponding to distances from 60 nm to the nm, together with the best fit, obtained by a bilayer form factor with half thickness of about 3.6 nm, with the hydrophobic and hydrophilic portions of 1.6 nm and of 2 nm, respectively. The thickness of the hydrophobic core of the bilayer is not affected by the presence of HA-Chol molecules. Rather, the contrast profile of the vesicle changes mainly in the external hydrophilic shell, which is in agreement with the presence on the niosome surface of hyaluronic acid chains.

**Figure 5:**
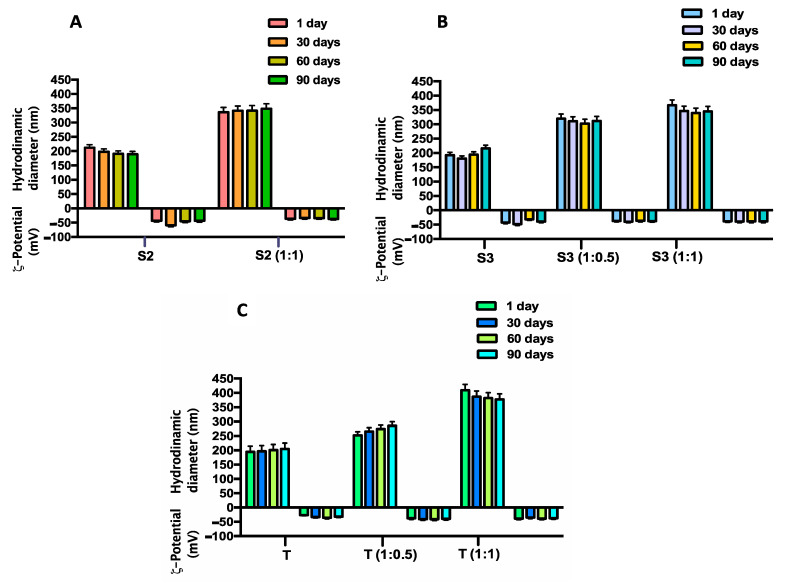
Time stability studies in terms of hydrodynamic diameter and ζ-potential variations of uncoated and of HA-Chol-coated niosomes up to 90 days at 25 °C temperature of storage. Panel (**A**) sample S2, uncoated and coated (S2 1:1) ; Panel (**B**) sample S3, uncoated and coated (S3 1:0.5; S3 1:1); Panel (**C**) sample T, uncoated and coated (T 1:1).

Stability studies carried out on coated and uncoated vesicles, underline a good stability of selected Span and Tween vesicles also in presence of HA-Chol throughout the time interval analyzed and at the storage temperature of 25 °C (Figure 5). The same results were obtained at the storage temperature of 4 °C (data not shown).

**Figure 6:**
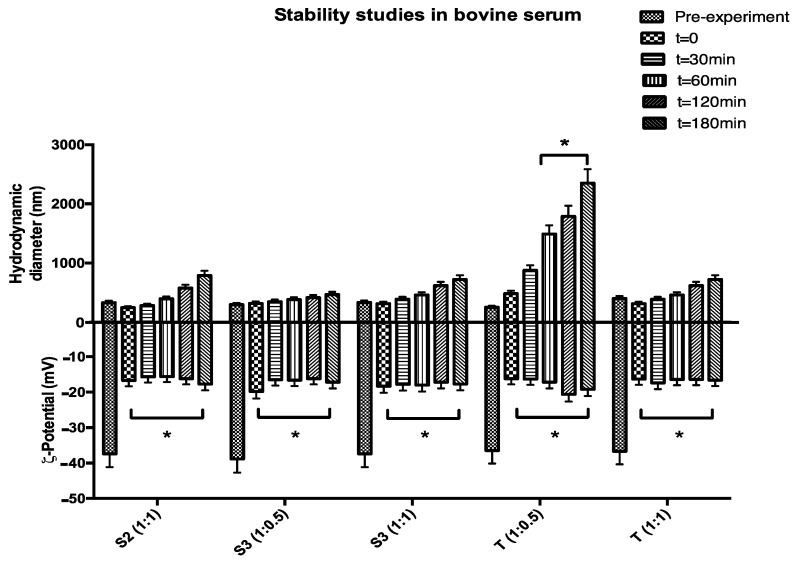
Biological stability studies in terms of hydrodynamic diameter and ζ-potential variations of HA-Chol-coated Span (S2 and S3) and Tween (T) niosomes up to 3 h at 37 °C (* *p* < 0.05).

Biological stability of HA-Chol-coated vesicles was determined in presence of bovine serum evaluating the hydrodynamic diameter and ζ-potential variations up to 3 h at body temperature (37 °C). As shown in Figure 6, all analyzed samples show good stability in hydrodynamic diameter except for sample T (1:0.5), highlighting that in the case of Tween niosomes a higher HA-Chol concentration is required to assure stability. This is probably dependent on the PEG coating typical of Tween niosomal surface, which to some extent could make it difficult for the HA-Chol to move closer to the niosomal surface. Moreover, the presence HA coating, is able to significantly decrease human serum protein adsorption because it exhibits negative charge densities able to reduce nanocarrier–protein interaction [14]. HA coating presentation is a tool to modulate and control the receptor-mediated uptake of HA-coated nanoparticles [28].

ζ-potential values show an immediate and significative reduction probably due to an immediate niosomal surface coating by counterions present in the bovine serum medium (Figure 6); this interaction is partially decreased by HA coating but not blocked at all. HA was mainly adsorbed at the polar surface of the surfactant bilayers, with the Chol hydrophobic segment partially inserted between aliphatic chains. Moreover, Tween-based niosomes were also able to interact with HA through hydrogen bonding when it assembled to form colloidal objects. Therefore, hydrophobic interactions and hydrogen bonds were likely to be the predominant mechanism of HA-Chol coating in HA-Tween-based niosomes [29]. Plasma stability studies, reported in Figure 6 are preliminary ones, because the role of protein corona interaction needs to be better defined and characterized in order to understand, not only physical chemical variations of the proposed structure, but also the biological feature variations.

**Figure 7:**
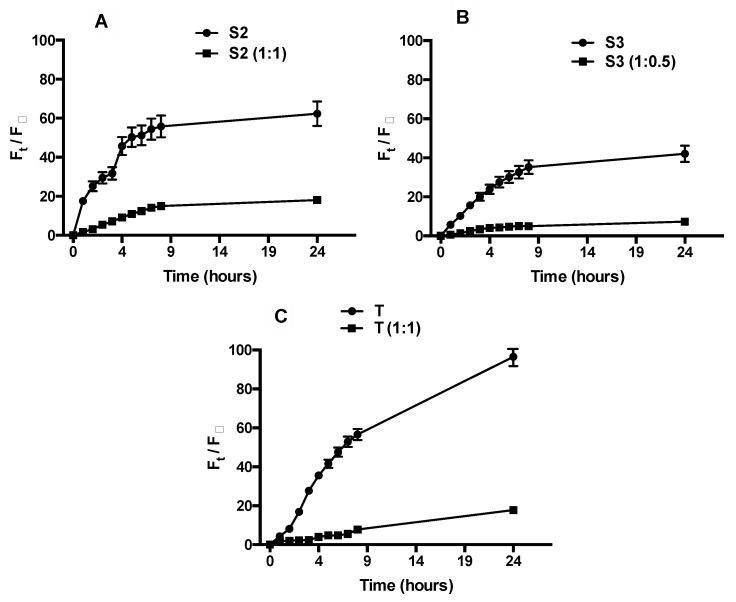
Release profile of calcein by uncoated and HA-Chol-coated Span (panel (**A**) S2; Panel (**B**) S3) and Tween (Panel (**C**) T) niosomes expressed as fluorescence percentage of calcein released.

Calcein release is higher for Tween niosomes due to their inner structure: unilamellar vesicles with a more fluid bilayer and a more hydrated vesicular surface because of the presence of PEG units (Figure 7). In presence of HA-Chol, a strong reduction in probe release is shown.

This behavior is confirmed for all Span samples. In uncoated ones, the calcein release is smaller than Tween niosomes, probably because of the presence of a multilamellar structure, as evidenced by SAXS analyses.

In presence of HA-Chol the release is extremely reduced because of an increase of steric hindrance responsible for probe release reduction.

The delayed and decreased calcein release by HA-Chol-coated samples is of particular interest, because samples, once administered, must reach their target without losing the entrapped material and only after the receptor binding, cell internalization takes place via receptor-mediated endocytosis and the entrapped material will be released inside the cell.

**Figure 8:**
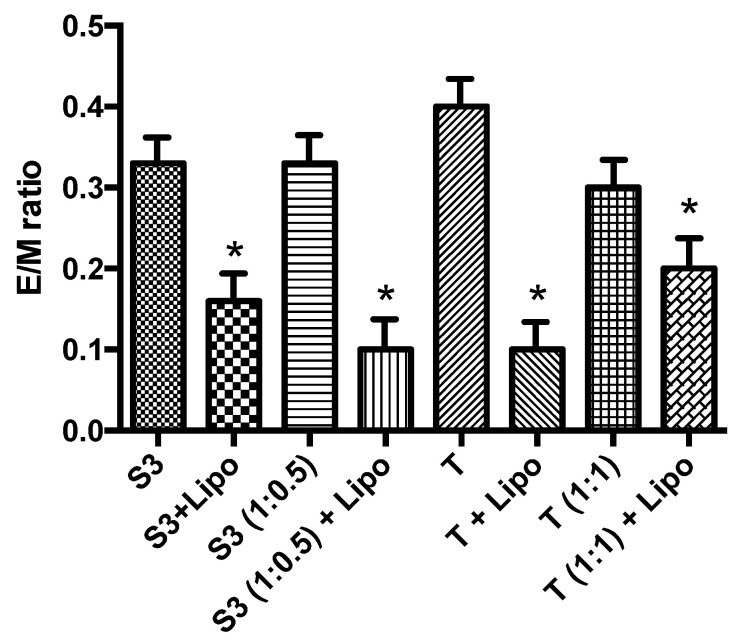
Pyrene E/M ratio decrease. Reported data represent the mean of three experiments ± S.E. **p ≤ 0.01 compared to labelled vesicles in absence of liposomes (* *p* < 0.05).

Vesicle internalization by endocytosis pathway, probably due to HA coating is fundamental to obtaining vesicle internalization, so the overall interaction with membrane lipid behavior can be of interest to have an idea of uncoated and coated vesicles behavior in vivo. Phosphatidylcholine constitutes one of the main components of endosome membranes. In a first attempt to elucidate the fusogenic capability of HA-coated niosomes with the liposomal membrane, the lipid mixing experiments were carried out by fluorescence spectroscopy, using Pyrene dispersed in the surfactant vesicle bilayer as a probe. Membrane interaction was indicated by a decrease in the E/M ratio due to redistribution (i.e., dilution) of the fluorescent probe over the available lipid phase (Figure 8): E/M decrease is evident for all samples, corresponding to an effective dilution of the probe. In addition, as shown in Figure 8 the lipid mixing is clearly dependent on cholesterol content in vesicle bilayer, in fact, as already demonstrated [30] cholesterol induces membrane lipid ordering and resists the incorporation of surfactants and alkanols) into PC membranes, which is because T sample with higher Chol content shows a lower E/M ratio reduction.

The generally accepted membrane interaction mechanisms share in common the requirement for initial close apposition of the fusing membranes and the formation of highly curved non-bilayer intermediates. HA coating is more evident in hydrophilic bilayer and this prevents both the aggregation and the formation of non-bilayer intermediates [30].

## 3. Materials and Methods

### 3.1. Materials

Tween 20 (polysorbate 20 or Polyoxyethylenesorbitan monolaurate), Span 20 (sorbitan monolaurate), cholesterol (Chol), Hepes salt (Sodium 2-(4-(2-hydroxyethyl)piperazin-1-yl)ethanesulfonate), Sephadex G-75, Pyrene, 1,6-diphenyl-1,3,5-hexatriene (DPH), calcein, bovine serum, 4-bromobutyric acid, N-methyl 2-pyrrolidone (NMP), *N*-(3-dimethylaminopropyl)-*N*’-(ethylcarbodimide hydrochloride) (EDC∙HCl), and 4-(Dimethylamino)pyridine (DMAP) were purchased from Sigma-Aldrich. Hyaluronan sodium salt (HANa, Mw = 2.2 × 10^5^) was provided by Contipro (Dolní Dobrouč, Czech Republic) and it was modified in hyaluronan tetrabutylammonium salt (HATBA) form using a Dowex cation exchange resin. 1,2-dimyristoyl-sn-glycero-3-phosphocholine (DMPC) was obtained from Avanti Polar Lipids (Alabaster, AL, USA). All other products and reagents were of analytical grade.

### 3.2. Methods

#### 3.2.1. Preparation and Purification of Niosomes

The thin-film hydration method was used in order to prepare niosomal vesicles [5]. Two different non-ionic surfactants, Span20^®^ or Tween20^®^, were employed and mixed with cholesterol in the molar ratio shown in the Table 4. Both components were solubilized with an organic mixture (CH_3_Cl:CH_3_OH, 3:1 *v*/*v*) and then evaporated by a rotary vacuum evaporator in order to obtain a “film.” Hepes buffer (0.01 M, pH 7.4) was added to the dried film and then vortexed to obtain multilamellar vesicles. Then, the suspension is sonicated for 5 min, at 60 °C with 18% of amplitude for Span20^®^ niosomes and 16% for Tween20^®^ niosomes, by using a microprobe operating at 20 kHz (VibraCell-VCX 400-Sonics, Taunton, MA, USA). Finally, gel permeation chromatography was carried out to purify the suspension by using Sephadex G75 column (glass column of 50 × 1.2 cm) with Hepes buffer as eluent.

#### 3.2.2. Synthesis of Hyaluronic Acid Derivatized with Cholesterol 

The HA-Chol derivative was obtained by a slight modification of a method previously described [15]. Briefly, cholesterol was previously derivatized with 4-bromo-butyric acid in the presence of EDC∙HCl and DMAP in order to obtain a bromo-butyric derivative of cholesterol (Chol-Br); then, HATBA (200 mg) was dissolved in *N*-methyl-2-pyrrolidone (NMP) (10.0 mL) and Chol-Br (16 mg, corresponding to 10% mol_Chol_/mol_HA_ repeating units) solubilized in 2 mL of NMP was added. The reaction was kept under magnetic stirring for 48 h at 38 °C and then 2 mL of NaCl saturated solution was added dropwise, to allow the replacement of TBA^+^ with Na^+^ ions. The product was then extensively dialyzed against distilled water (Visking tubing, cut-off: 12,000–14,000) and the HA-Chol derivative was finally recovered by freeze-drying (yield: 80%).

#### 3.2.3. Coating of Niosomes with HA-Chol Derivative

Two solutions of HA-Chol with different concentrations were tested, 1 mg/mL (Table 5) or 1.5 mg/mL (Table 6), in Hepes buffer up to the molar ratio shown in both tables. HA-Chol solutions were added to the niosomal dispersion in equal *v*/*v* ratio (niosomal dispersion/HA-Chol solution). The resulting preparations were stirred up to 24 h at room temperature, measuring Z-average and ζ-potential at different time points to have an idea of the functionalization progression.

#### 3.2.4. Solid State NMR 

The ^13^C CP-MAS NMR spectra of the polymer HA-Chol and of the coated samples S2 (with Span 20:HA-Chol in the ratio 1:1) and T (with Tween 20:HA-Chol 1:0.5) were performed at the “Annalaura Segre” NMR Laboratory of Institute of Chemical Methodologies of CNR of Montelibretti (Rome, Italy) on a Bruker AVANCE III spectrometer operating at 200.63 MHz.

The lyophilized samples were introduced into a cylindrical ZrO_2_ rotor (d = 4 mm) with spherical inserts, sealed with Kel-F caps and spun at a speed-rate of 8 kHz. The spectra were obtained using a contact time for the cross polarization of 1.5 ms with a recycle delay of 2 s.

Spectra were acquired using 1024 data points in the time domain, zero filled and Fourier transformed. The chemical shift was externally referred to tetramethylsilane (TMS).

For each sample 4000 scans were acquired in the range 0–220 ppm. 

#### 3.2.5. Dynamic Light Scattering and ζ-Potential Measurements

Hydrodynamic diameter and size distribution (polidyspersity index, PDI) of all samples were measured at 25 °C by using a Zetasizer Nano ZS90 (Malvern Instruments, Malvern, UK), equipped with a 5 mW HeNe laser, λ = 632.8 nm. Before the measurements, all samples were diluted 100 times with Hepes buffer in order to avoid multiple scattering phenomena.

The scattering angle was 90° and the analysis of the intensity autocorrelation function was carried out using Contin algorithm [31]. The calculated mean hydrodynamic radius corresponds to the intensity weighted average [32].

Electrophoretic mobility of the vesicles was measured by laser Doppler anemometry using the Malvern Zetasizer Nano ZS90 apparatus. The mobility (u) was converted in ζ-potential using the Smoluchowski relation ζ = uη/є, where η is the viscosity and є the permittivity of the solvent phase [33]. 

#### 3.2.6. Transmission Electron Microscopy (TEM)

The morphology of uncoated and HA-coated niosomes was visualized by TEM. One drop of the selected samples was placed onto a formvar carbon-coated grid and allowed to dry to a thin film. Before complete drying of this film on the grid, it was negatively stained with 2% filtered aqueous sodium phosphotungstate. After removing of the excess dye by a filter paper, the samples were air dried and examined by a FEI 208S transmission electron microscope (FEI Company, Hillsboro, OR, USA) with an accelerating voltage of 80 kV.

#### 3.2.7. Stability Studies 

Time stability of Nio and HA-Chol-Nio was examined at 4 °C and room temperature for 90 days in order to assess the colloidal stability. Periodically each sample was analyzed by measuring hydrodynamic diameter and ζ-potential at definite time intervals (1, 30, 60, and 90 days).

The biological stability of niosomes with and without HA in presence of bovine serum (BS) was evaluated. A mixture of Nio or HA-Chol-Nio and 45% of BS was prepared and placed at 37 °C. By DLS, the mix was analyzed at different time intervals (0, 0.5, 1, 2, and 3 h), evaluating the particle size and ζ-potential.

#### 3.2.8. Small Angle X-ray Scattering

SAXS technique was applied to obtain information on the internal structure of niosomes [34,35].

Measurements were carried out at the high brilliance ID02 beamline at the ESRF Synchrotron (Grenoble, France) at 25 °C. The excess scattered intensity of the samples in the region of momentum transfer 0.08 nm^−1^ < q < 5 nm^−1^ was obtained after the subtraction of the background and the buffer contributions. The intensity profile of niosomes was modelled to a multi-shell hollow sphere (SasView 4.2.1 software [36]).

#### 3.2.9. Bilayer Characterization

In order to evaluate the influence of HA-Chol coating on niosomal bilayer, two fluorescent probes were employed: DPH and Pyrene.

In particular, fluorescence anisotropy studies were carried out to determinate the bilayer fluidity. DPH-loaded niosomes were evaluated by adding a DPH solution to niosomal components at the final concentration of 2 × 10^−4^ M, before adding the organic mixture. Fluorescent measurements were carried out with a Perkin-Elmer LS50B spectrofluorimeter at excitation and emission wavelengths of 400 nm and 425 nm, respectively. The fluorescence anisotropy of the all formulations was calculated according to the equation proposed by Lentz et al. [37].

The fluorescence experiments on Pyrene-loaded niosomes were carried out to evaluate the polarity and microviscosity of the bilayer in the vesicles. The samples were prepared by adding Pyrene (4 mM) to the niosomal components (same preparation method as above). By fluorescence measurements it is possible to investigate the lateral distribution and the mobility of the membrane compounds. Pyrene is a florescence probe, with a spectrum characterized by five emission peaks as monomer (from I_1_ to I_5_) and one as excimer (I_E_). The monomer and the excimer have different fluorescence signals and the ratio between the several fluorescence intensities is directly related to the probe distribution in the bilayer. In particular, the ratio I_1_/I_3_, corresponding to the first and third vibration bands of Pyrene spectrum, is related to the polarity of the probe environment. Pyrene can form intramolecular excimer based on the viscosity of the probe microenvironment [38].

#### 3.2.10. Interaction Properties of Nanovesicles with Liposomes

In order to demonstrate the ability of niosomes to interact with cells, to deliver their cargo into the cell, liposomal vesicles were employed in order to mimic the cell membrane.

Liposomes composed by DMPC (6.7 mM) and Chol (2.6 mM) were prepared by thin layer evaporation method previously described for niosome preparation. Pyrene-loaded niosomes with or without HA-Chol were added to liposomes in a 1:1 ratio and the obtained complex dispersion was stirred up to 3 h at 37 °C. The interaction between niosomes and liposomes was evaluated by fluorescence spectroscopy method as reported by Polozova and Winnik [30,39].

#### 3.2.11. Calcein Release Studies

Calcein release profiles of niosomes with and without HA-Chol coating were evaluated by using the dynamic dialysis bag method [40]. Calcein loaded-niosomes were prepared as previously described; in particular during the hydration, a calcein solution (a calcein self-quenched solution: 0.01 M of calcein in Hepes buffer) was used. Then, each sample was separately loaded into dialysis bags (molecular weight cut-off: 8000 MW by Spectra/Por^®^), which were immersed in Hepes buffer at 37 °C and shaken by magnetic stirring. At determined time points (each hour from 0 to 8 and 24 h), the medium was withdrawn, analyzed, and replaced in the medium to keep the sink condition.

The calcein release from the niosomes was measured by following the probe dequenching by using a Perkin-Elmer LS50B spectrofluorometer with a λ_ex_ and λ_em_ of 492 nm and 520 nm, respectively.

The release percentage (F%) was calculated by the ratio between the fluorescence read at the specific time point the reference value correlated to the total calcein amount entrapped into the niosomes, which was measured after vesicle disruption with isopropyl alcohol (1:1, *v*/*v*).

Furthermore, in order to evaluate the vesicle release profile in presence of biological fluids, at each sample loaded with calcein was added 45% of bovine serum. The calcein release profile was measured as previous reported [27].

#### 3.2.12. Statistical Analysis

Statistical data analysis was performed using the Student’s *t*-test. The difference between different data group was considered significant when the *p*-value was less than 0.05.

## 4. Conclusions

Niosomes made by Tween 20 and Span 20 can be efficiently coated by HA-Chol. Cholesterol derivatization allows for a stable anchoring of the HA molecule on the niosomal surface, as demonstrated by the different characterization techniques. Details of coating depend on the initial concentration of HA-Chol. In our prodromic cell-free interaction studies, the HA-Chol concentration appears to modulate the niosome-cell-mime interaction, namely, interaction with the cell-mime membrane is favored when the initial HA-Chol content of niosomes is relatively low. Further focused cell-supported studies will address the stable HA-Chol-niosomes in vivo, their selectivity for CD44 bearing cells and their bioavailability.

## Figures and Tables

**Table 1:** DLS analyses of uncoated Span and Tween niosomes. Values represent the mean ± standard deviation of *n* = 3 sample measurements.

Sample	Span20 (mM)	Tween20 (mM)	Chol (mM)	Hydrodynamic Diameter (nm) ± SD	ζ-Potential (mV) ± SD	PDI
S1	15.0	-	15.0	98.0 ± 2.0	−34.9 ± 0.1	0.2
S2			11.2	212.0 ± 2.0	−43.3 ± 2.5	0.2
S3			7.5	192.0 ± 2.0	−42.3 ± 2.5	0.2
T	-	15.0	15.0	194.0 ± 2.0	−26.7 ± 2.4	0.2

**Table 2:** DLS analyses of HA-Chol-coated Span and Tween niosomes. Values represent the mean ± standard deviation of *n* = 3 sample measurements.

Sample with HA-Chol	Hydrodynamic Diameter (nm) ± SD	ζ-Potential (mV) ± SD	PDI
S2 (1:1)	331.8 ± 6.0	−37.4 ± 2.3	0.6
S3 (1:0.5)	297.6 ± 19.3	−36.8 ± 1.7	0.6
S3 (1:1)	333.9 ± 14.1	−37.4 ± 0.6	0.6
T (1:0.5)	250.5 ± 12.9	−36.5 ± 0.9	0.7
T (1:1)	401.4 ± 16.8	−36.7 ± 1.3	0.4

**Table 3:** Bilayer characterization by fluorimetric assays of uncoated and HA-Chol-coated niosomes utilizing two probes: pyrene for microviscosity and polarity assessment and DPH for fluidity evaluation.

Sample with and without HA-Chol	Fluidity (Anisotropy)	Polarity (I_1_/I_3_)	Microviscosity (I_E_/I_3_)
S2	0.11	1.14	0.38
S2 (1:1)	0.24	1.16	0.30
S3	0.14	1.12	0.74
S3 (1:0.5)	0.15	1.23	0.36
S3 (1:1)	0.21	1.22	0.33
T	0.20	1.58	0.18
T (1:0.5)	0.27	1.15	0.17
T (1:1)	0.26	1.12	0.29

**Table 4:** Sample compositions.

Sample	Span20 (mM)	Tween20 (mM)	Chol (mM)
S1	15.0	-	15.0
S2			11.2
S3			7.5
T	-	15.0	15.0

**Table 5:** Coating ratios between sample and HA-Chol 1mg/mL.

Sample	Molar Ratio
S3	1:0.5
	1:1
T	1:0.5

**Table 6:** Coating ratios between sample and HA-Chol 1.5 mg/mL.

Sample	Molar Ratio
S2	1:1
T	1:1

## Data Availability

Not applicable.

## Sample Availability

Samples of the compounds are not available from the authors.
